# Diagnostic and Predictive Value of Using RGD PET/CT in Patients with Cancer: A Systematic Review and Meta-Analysis

**DOI:** 10.1155/2019/8534761

**Published:** 2019-01-10

**Authors:** Jie Liu, Shuanghu Yuan, Linlin Wang, Xindong Sun, Xudong Hu, Xue Meng, Jinming Yu

**Affiliations:** ^1^School of Medicine and Life Sciences, University of Jinan-Shandong Academy of Medical Sciences, Jinan, Shandong 250022, China; ^2^Department of Radiation Oncology, Shandong Cancer Hospital and Institute-Shandong Cancer Hospital Affiliated to Shandong University, No. 440 Jiyan Road, Jinan 250117, Shandong, China; ^3^Shandong Academy of Medical Sciences, Jinan 250022, Shandong, China

## Abstract

The purpose of this study was to assess the diagnostic value of arginine-glycine-aspartic acid (RGD) PET/CT for tumor detection in patients with suspected malignant lesions and to determine the predictive performance of RGD PET/CT in identifying responders.* Methods*. The PubMed (Medline), EMBASE, Cochrane Library, and Web of Science databases were systematically searched for potentially relevant publications (last updated on July 28th, 2018) reporting the performance of RGD PET in the field of oncology. Pooled sensitivities, specificities, and diagnostic odds ratios (DORs) were calculated for parameters. The areas under the curve (AUCs) and Q⁎ index scores were determined from the constructed summary receiver operating characteristic (SROC) curve. We explored heterogeneity by metaregression.* Results*. Nine studies, five including 216 patients that determined diagnostic performance and three including 75 patients that determined the predictive value of parameters, met our inclusion criteria. The pooled sensitivity, pooled specificity, DOR, AUC, and Q⁎ index score of RGD PET/CT for the detection of underlying malignancy were 0.85 (0.79-0.89), 0.93 (0.90-0.96), 48.35 (18.95-123.33), 0.9262 (standard error=0.0216), and 0.8606 for SUVmax and 0.86 (0.80-0.91), 0.92 (0.88-0.94), 40.49 (14.16-115.77), 0.9312 (SE=0.0177), and 0.8665 for SUVmean, respectively. The pooled sensitivity, pooled specificity, DOR, AUC, and Q⁎ index score of RGD PET/CT for identifying responders were 0.80 (0.59-0.93), 0.74 (0.60-0.85), 15.76 (4.33-57.32), 0.8682 (0.0539), and 0.7988, respectively, for SUVmax at baseline.* Conclusion*. The interesting but preliminary data in this meta-analysis demonstrate that RGD PET/CT may be an ideal diagnostic tool for detecting underlying malignancies in patients suspected of having tumors and may be able to efficiently predict short-term outcomes.

## 1. Introduction

Angiogenesis, the process of new blood vessel formation from preexisting vasculature, is recognized as a key mechanism involved in tumor growth, invasion, and metastasis [1]. In 1971, Judah Folkman first stressed that a tumor could not grow larger than a few millimeters in diameter without angiogenesis occurring to transport nutrients and oxygen [2, 3]. The vascular effects of antitumor therapy on microvessel density may precede the impact on tumor size by a long interval, especially when the tumor volume cannot change significantly [4].

Integrin-mediated cell adhesion plays an important role in many essential normal cellular and pathological functions [5]. Integrin *α*v*β*3, which has been widely studied, is significantly upregulated in tumor cells and activated endothelial cells but not in resting vessel cells in normal regions [6]. Therefore, imaging integrin *α*v*β*3 expression may be valuable for diagnosis and assessing suitable patients for particular treatments. Imaging of various tripeptide RGD sequence-containing integrins has been extensively evaluated because of their high affinity and specificity with regard to integrin *α*v*β*3. Compared to other methods of imaging *α*v*β*3, the PET/CT approach is likely to be widely applied in tumor patients because of its high sensitivity to low amounts of tracer and its unlimited depth penetration [7]. Indeed, there have been great efforts to develop radiolabeled RGD peptides, and ^18^F, as a radioisotope for labeling peptides, is commonly used because its half-life is suitable for routine clinical use [8]. In addition, ^18^F-Galacto-RGD [9–14], ^18^F-Fluciclatide-RGD [15, 16], ^68^Ga-NOTA-PRGD2 [17], ^18^F-Alfatide [18], ^18^F-FPPRGD2 [19], and ^18^F-Alfatide II [20, 21] have been under clinical investigation for their relevance to the diagnostic ability of RGD PET/CT, but only sensitivity has been studied to date. Among them, ^18^F-Alfatide II and ^68^Ga-NOTA-PRGD2 show advantages over the others in terms of easy preparation, fast labeling, and in vivo pharmacokinetics [22]. The results of the majority of the studies have demonstrated positive potential, though some studies have had small sample sizes. To further evaluate the diagnostic ability of RGD PET/CT, we conducted a meta-analysis of the clinical literature to obtain data regarding the sensitivity, specificity, positive likelihood ratio (PLR), and negative likelihood ratio (NLR).

Even when treated by the standard therapy of concurrent chemoradiotherapy (CCRT), one-third of patients with advanced non-small cell lung cancer (NSCLC) still experience local treatment failure [23]. Thus, finding an effective prediction tool to select patients likely to respond to therapy would help guide individualized treatment. Although morphologic treatment effects cannot be detected by conventional imaging techniques at early time points but only after several weeks or months [24, 25], imaging tumor angiogenesis may be used for patient risk stratification before starting therapy [22]. Therefore, we performed a meta-analysis to evaluate the role of RGD PET/CT in predicting the short-term outcomes of therapy among NSCLC patients.

## 2. Methods

### 2.1. Literature Search Strategy

All relevant articles were retrieved from the PubMed (Medline), EMBASE, Cochrane Library, and Web of Science databases. The databases were searched based on the following keywords and text words: (“RGD” OR “alfatide”) and (“PET” OR “positron emission tomography”) and (“neoplasms” OR “tumor” OR “cancer” OR “neoplasm” OR “tumour”). The search was last updated on July 28th, 2018. There were no beginning date restrictions.

### 2.2. Inclusion and Exclusion Criteria

Published articles were selected according to the following inclusion criteria: (1) original articles revealing the performance of RGD PET or PET/CT for the diagnosis of neoplasms or predicting the efficacy of treatment; (2) studies in which the final diagnoses of patients were confirmed by histopathology or comprehensive assessment containing clinical and radiologic follow-up; (3) studies in which RGD PET or PET/CT was used as the single reference standard for neoplasm diagnosis; (4) studies in which the short-term efficacy of cancer therapy was based on tumor regression grading (TRG), response evaluation criteria in solid tumors (RECIST), or changes in the volumes of residual lesions on MRI; (5) studies in which RGD PET or PET/CT was performed at baseline or during therapy for the prediction of a curative effect; (6) articles with sufficient data to acquire true positive (TP), false positive (FP), false negative (FN), and true negative (TN) rates; (7) articles published in English. The exclusion criteria included the following: (1) duplicate studies; (2) letters, case reports, reviews, comments, and meeting abstracts; (3) laboratory studies, animal studies, or studies unrelated to the diagnosis or prediction of cancer; (4) studies with patients who had been treated before for diagnostic purposes; (5) studies that were not related to the prediction of short-term outcomes due to inconsistent long-term outcomes and too few studies.

### 2.3. Data Extraction

The following data were extracted from the studies: basic information of the studies (names of the first authors, country of origin, year of publication, and study design), population characteristics (number of subjects or lesions, sex distribution, and age distribution), technical aspects (imaging methods, parameters, or PET technique), the effect index (the TP, FP, FN, and TN rates for the PET imaging), and methods of tumor determination or response criteria.

### 2.4. Quality Assessment of Included Studies

Two reviewers independently assessed the quality of the included studies. Studies testing the diagnostic value of RDG PET or PET/CT were assessed by QUADAS-2. The scale consists of the following four domains: patient selection, index test, reference standard and flow, and timing. Each section includes assessment of the risk of bias (“low”, “high”, or “unclear”) and the applicability of diagnostic accuracy [29]. Studies investigating the predictive value of RDG PET or PET/CT were evaluated using the Newcastle-Ottawa Scale (NOS). The scale involves the following three items: subject selection criteria, comparability of subjects, and outcomes. High-quality articles have total scores of more than 5 points, with a maximum total score of 9 points [30].

### 2.5. Statistical Analysis

Pooled estimates of the sensitivity, specificity, and DOR with 95% confidence intervals (CIs) were determined based on bivariate analysis of patients or lesions. We evaluated heterogeneity among the studies through the likelihood ratio I^2^ index. We assigned categories of low, moderate, and high heterogeneity to I^2^ values of 25%, 50%, and 75% [31]. The DerSimonian-Laird method (random effect model) was applied for the meta-analysis if heterogeneity existed; otherwise, the Mantel-Haenszel method (fixed effects model) was employed. Summary receiver operating characteristic (SROC) curves with the AUC and the Q^*∗*^ estimate were obtained. We analyzed the diagnostic abilities of the four parameters and heterogeneity by metaregression. Publication biases were assessed by Deek's funnel plots. The main analyses were performed in Meta-Disc 1.4.

## 3. Results

### 3.1. Search Results

**Figure 1:**
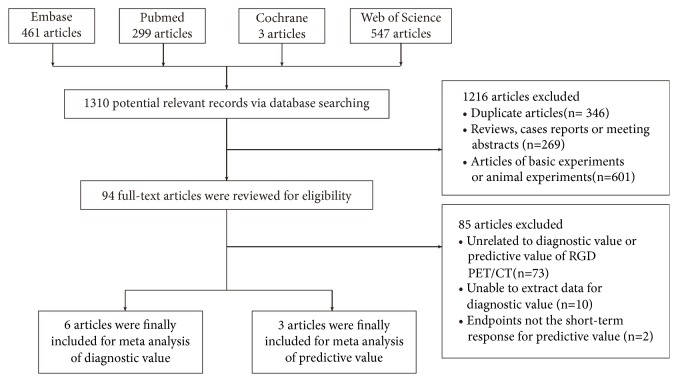
Flow diagram of the selection process of eligible studies.

Using a previously established retrieval strategy, we initially identified 1310 relevant papers from the databases, as follows: 461 in EMBASE, 299 in PubMed, 3 in the Cochrane Library, and 547 in Web of Science. In total, 1216 manuscripts were excluded for the following reasons: 346 were duplicates; 269 were reviews, case reports, or meeting abstracts; and 601 were about basic experiments or animal experiments. The remaining 94 articles were subjected to further full-text assessment. After careful reading, 85 of the papers were excluded for the following reasons: 73 were unrelated to the diagnostic or predictive value of RGD PET/CT; 10 lacked sufficient data to acquire or calculate TP, FP, FN, and TN rates; and 2 had endpoints that were not short-term responses. Ultimately, 9 studies were included. Six articles with 9 sets of data and a total of 216 patients were eligible for inclusion to assess the diagnostic value of RGD PET/CT, and 3 articles with 75 patients were suitable for evaluating the predictive value of RGD PET/CT (Figure 1) [8, 26–28, 32–35].

### 3.2. Characteristics of the Included Studies and Quality Assessment

The main characteristics of the studies are shown in Table 1. Three of the selected articles each diagnosed both the primary tumor and the lymph node metastasis status of patients with lung cancer, one of which also examined the diagnostic potential of ^68^Ga-Alfatide II PET/CT in differentiating between NSCLC and tuberculosis patients. Six studies used different RGD radioligands. We selected the number of lesions or metastases for analysis rather than the number of patients when both were counted in the studies. Several parameters (SUV_max⁡_, SUV_mean_, tumor-to-normal tissue ratios, and visual analysis) were used as diagnostic parameters. Eight sets of data were selected for SUV_max⁡_, five sets of data for SUV_mean_, three sets of data for visual analysis, and only one set of data for tumor/blood ratios (T/N). Tumor lesions or metastases were all finally diagnosed by histopathology. The patient-based diagnostic parameters of RGD PET/CT for the studies are shown in Table 2. The diagnostic sensitivity ranged from 75.00% to 100%, and the diagnostic specificity ranged from 44.44% to 100%.

The main characteristics of the three studies that investigated the prediction of short-term effects are presented in Table 3. These studies enrolled patients with locally advanced rectal cancer, GBM after surgical resection, and advanced NSCLC. The parameter was SUVmax before CCRT. Xiaohui Luan et al. investigated the predictive value of multiple parameters, including SUVpeak and T/N. Overall, short-term response criteria differed according to tumor type. The predictive parameters of RGD PET/CT from the studies are listed in Table 4. The prediction sensitivity ranged from 73% to 100%, and the prediction specificity ranged from 66.7% to 88.9%.

**Figure 2:**
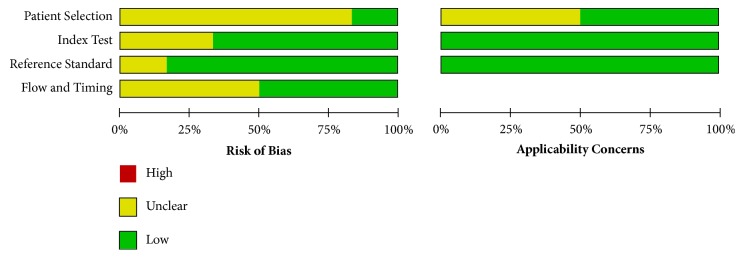
Quality Assessment of Diagnostic Accuracy Studies-2 was used to assess the quality of the studies for diagnostics.

The quality of the studies that investigated diagnostic potential was assessed by QUADAS-2, and these studies were determined to have a low risk of bias (Figure 2). The quality of the studies that investigated the prediction of short-term outcomes was assessed by the NOS, with scores ranging from 7 to 8; this result indicates that the studies were of high quality (Table 5). There was no overlap among the cohorts enrolled in the articles. In addition, all the included studies were prospective.

### 3.3. Heterogeneity and Threshold Effect Assessment

Heterogeneity among the studies was determined using the Chi-square test. There was clear heterogeneity in the sensitivity and specificity of the parameters for diagnosis, but there was no significant heterogeneity for the predictive value. Furthermore, there was no threshold effect among the studies (Table 6). Thus, the DerSimonian-Laird method (random effects model) was adopted to estimate pooled data for the diagnostic value, and the Mantel-Haenszel method (fixed effects model) was used for the predictive value.

### 3.4. Diagnostic Performance and Heterogeneity Analysis

**Figure 3:**
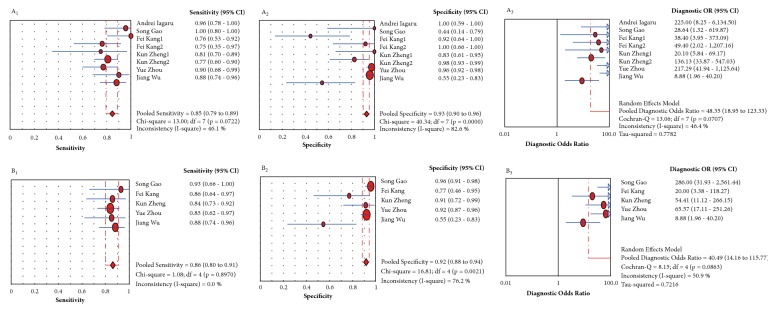
Forest plot of the sensitivity, specificity, and diagnostic OR (DOR) of RGD PET/CT for parameters (A1, A2, A3: sensitivity, specificity, and DOR for SUVmax; B1, B2, B3: sensitivity, specificity, and DOR for SUVmean) for the diagnosis of suspected carcinoma. Circle: likelihood ratios of individual studies. Diamond: pooled likelihood ratios of all five enrolled studies. Subscript 1: the set of data for the diagnosis of carcinoma in situ. Subscript 2: the set of data for the diagnosis of metastasis.

**Figure 4:**
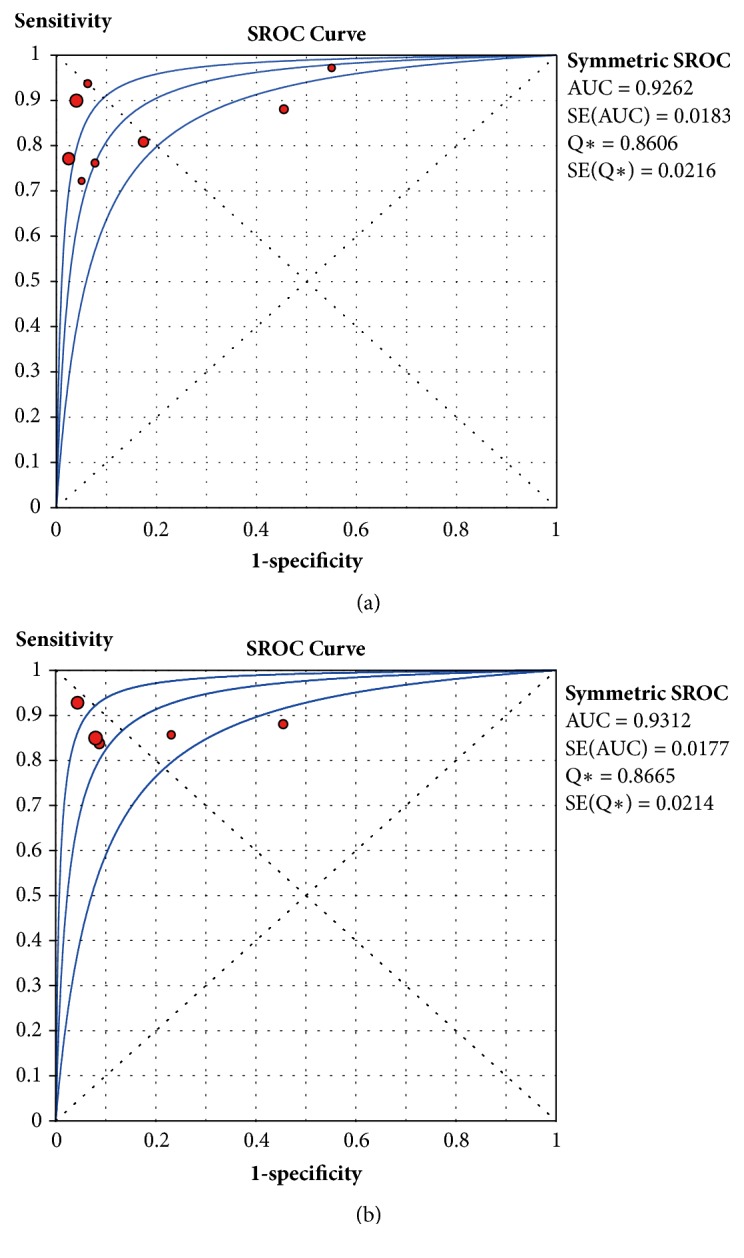
Summary receiver operating characteristic (SROC) curves of RGD PET/CT for parameters ((a): SROC curves for SUVmax; (b): SROC curves for SUVmean) for the diagnosis of suspected carcinoma. Circle: likelihood ratios of individual studies. The middle blue lines are the SROC curves, and the adjacent two lines are 95% confidence intervals. AUC: area under the ROC curve. SE: standard error. Q*∗*: Q index.

The pooled sensitivity, specificity, DOR, and SROC for SUVmax and SUVmean in diagnosing a primary tumor and lymph node metastasis are shown in Figures 3 and 4. This meta-analysis revealed a pooled sensitivity of 0.85 (0.79-0.89), specificity of 0.93 (0.90-0.96), and DOR of 48.35 (18.95-123.33) for SUVmax and a sensitivity of 0.86 (0.80-0.91), specificity of 0.92 (0.88-0.94), and DOR of 40.49 (14.16-115.77) for SUVmean for RGD PET/CT in patients with suspected tumors. Based on the SROC value of these parameters, we observed the AUC and Q*∗* of SUVmax to be greater than those of SUVmean (AUC=0.9262 SE=0.0216, Q*∗*=0.8606; AUC=0.9312 SE=0.9312, Q*∗*=0.9312). We then compared the diagnostic abilities of these four parameters, namely, SUVmax, SUVmean, T/N, and visual analysis, via regression analysis. The relative diagnostic odds ratio (RDOR) determined by meta-regression analysis was 0.79 (0.38-1.61), and there was no significant difference among the diagnostic abilities of the four parameters (P=0.4814).

### 3.5. Subgroup Analysis of Diagnostic Performance

To obtain further insight, we analyzed heterogeneity with regard to lesion location, RGD radioligand, and tumor type for SUVmax and for lesion location and RGD radioligand for SUVmean. However, no factors were found to clearly affect heterogeneity (Table 7).

### 3.6. Predictive Performance

**Figure 5:**
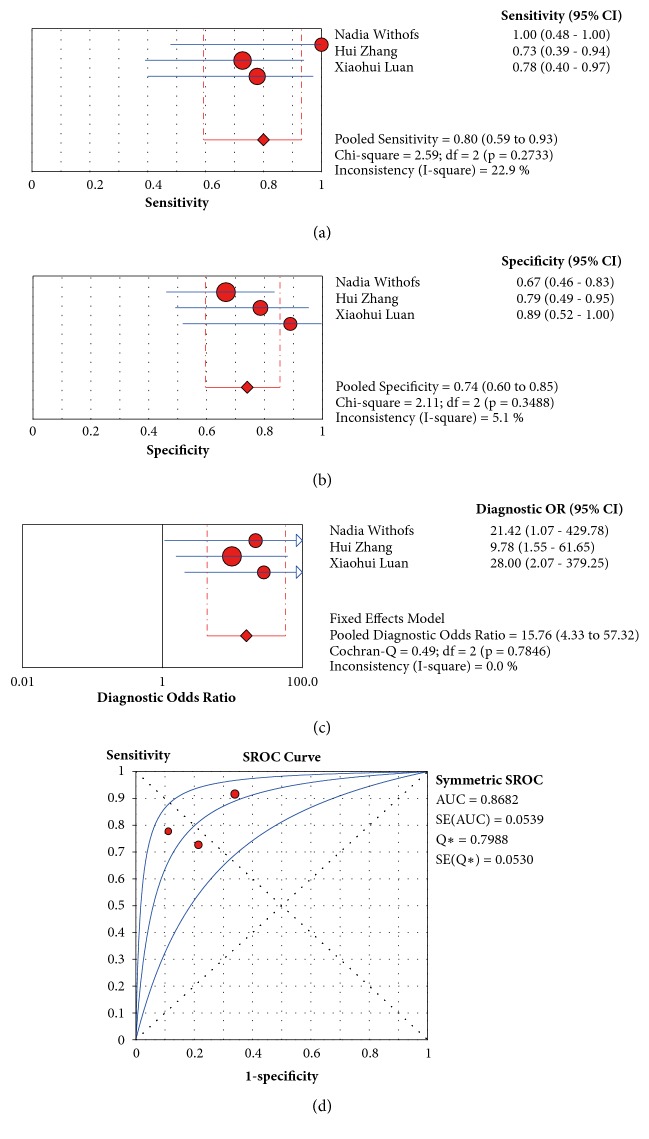
Forest plot of sensitivity, specificity, and DOR and the SROC curve of SUVmax at baseline of RGD PET for the prediction of short-term outcomes after treatment. Circle: likelihood ratios of individual studies. Diamond: pooled likelihood ratios of all three enrolled studies. The middle blue line is the SROC curve, and the adjacent two lines are 95% confidence intervals. AUC: area under the ROC curve. Circle: likelihood ratios of individual studies. Diamond: pooled likelihood ratios of all enrolled studies. The middle blue lines are the SROC curves, and the adjacent two lines are 95% confidence intervals. AUC: area under the ROC curve. SE: standard error. Q*∗*: Q index.

The pooled sensitivity, specificity, and DOR of SUVmax in the prediction of tumors were 0.80, 0.74, and 15.76 (4.33-57.32), respectively (Figure 5). The AUC and Q*∗* of SUVmax were 0.8682 and 0.7988, respectively.

### 3.7. Publication Bias

Due to the small number of studies that investigated other parameters, only studies involving SUVmax and SUVmean were tested for publication bias. Deek's funnel plots indicated no significant publication bias (P=0.474 for SUVmax and P=0.603 for SUVmean). We did not analyze publication bias for predictive studies because the number of included studies was relatively small.

## 4. Discussion

This is the first meta-analysis to evaluate the diagnostic performance of RGD PET/CT in the detection of underlying malignancy and to assess the ability of RGD PET/CT to predict the short-term effects of treatment. The tumor diagnostic ability for SUVmax was slightly superior to that of SUVmean, though there was no significant difference. There was considerable heterogeneity in SUVmax and SUVmean in the literature, and we have not yet reached a consensus regarding the factors influencing the diagnostic value of RGD PET/CT because of the limited number of studies included in the meta-analysis. Overall, the different receptor binding affinities and receptor-binding kinetics of different radiotracers may be an important factor. The inclusion of different tumor types in this meta-analysis may be another important reason for the observed inter-tumor heterogeneity; *α*v*β*3 expression level and tumor angiogenesis vary among tumor types. Meta-analysis of the use of the same RGD PET for the diagnosis of the same type of tumor is more persuasive [36].

As the receptor binding affinity and tumor retention of RGD radioligands improve, multiple types of RGD peptides are being used in clinical studies; ^18^F-Galacto-RGD and ^18^F-Fluciclatide were the first two such compounds. These two compounds have similar distribution characteristics, including heterogeneous tumor uptake in monitoring sensitivity for primary and metastatic foci. The sensitivity for evaluating primary lesions ranges from 83% to 100%, though the sensitivity of ^18^F-Galacto-RGD is only 33-54% for metastatic lymph nodes (LNs) and 46-78% for distant metastases [9–11, 13, 14, 16, 37]. Although the sensitivity of ^18^F-Fluciclatide was relatively better, reaching 88-94% for all lesions and 71-88% for metastases [15, 16], the moderate level of sensitivity for metastases is insufficient for tumor staging. As a result, to enhance the performance of integrin imaging, multimeric RGD peptides with increased receptor binding affinity and tumor retention have been used in clinical studies [38, 39]. The results showed that ^18^F-Alfatide PET/CT and ^18^F-FPPRGD2 PET/CT are able to clearly identify all primary lesions (100% sensitivity) [18, 19]; however, fewer primary lesions (83.8%) can be distinguished with ^68^Ga-NOTA-PRGD2 PET/CT [17]. We comprehensively compared the diagnostic ability of ^18^F-Alfatide II RGD PET/CT and ^68^Ga-NOTA-PRGD2 PET/CT using data from studies by Fei Kang and Kun Zheng. Although the detection rates for primary lesions and metastatic LNs were similar (76.92% versus 75% for primary lesions and metastatic LNs, respectively, in the study by Fei Kang; 80.88% versus 77.14% in the study by Kun Zheng), the specificity for metastatic LNs was slightly inferior to that for primary lesions (92.31% versus 100% in the study by Fei Kang; 82.61% versus 97.58% in the study by Kun Zheng, P>0.05) [33, 34]. ^18^F-Alfatide II PET/CT has been performed for diagnosing bone metastases [20] and brain metastases [21], and among different types of metastases, ^18^F-Alfatide II PET/CT demonstrates excellent diagnostic sensitivity for osteolytic metastases (100%), mixed bone metastases (100%), and mixed bone metastases (98%) but moderate sensitivity for osteoblastic metastases (70%). Regarding brain metastases, all 20 lesions from patients were identified by ^18^F-Alfatide II PET/CT. Yue Zhou et al. demonstrated a relatively higher sensitivity of ^18^F-Alfatide PET/CT for detecting NSCLC (90.0%) and squamous cell carcinoma (SCC) (100%) than adenocarcinoma (AC) (83.3%). This may be due to the low affinity of ^18^F-Alfatide for AC [26]. The much easier radiosynthetic procedure for ^18^F-Alfatide and ^18^F-Alfatide II compared to others would facilitate large-scale clinical trials [22]. Nonetheless, the diagnostic ability of RGD PET/CT still requires larger sample-size clinical trials for validation.

Overall, ^18^F-FDG PET/CT cannot completely replace invasive staging methods because of its relatively low specificity and high uptake by inflammatory LNs [40]. Compared with the 30.21% positive predictive value of ^18^F-FDG PET/CT, ^68^Ga-NOTA-PRGD2 PET/CT had a value of 90%[34]. FDG, as an analog of glucose, is transported into cells by glucose transporters (GLUTs) and phosphorylated by hexokinase. Similar to malignant cells, inflammatory cells exhibit increased expression of GLUT and increased affinity of GLUT toward deoxyglucose, leading to high uptake of FDG but not necessarily an increase in integrin *α*v*β*3 [34, 41, 42]. ^68^Ga-NOTA-PRGD2 PET/CT may be complementary to ^18^F-FDG PET/CT because of its lower sensitivity but higher specificity in the diagnosis of tumors. However, in the study by Song Gao et al., uptake by benign lesions in nine patients was heterogeneous [8]. Jiang Wu et al. also found lower specificity for ^18^F-Alfatide II PET/CT (54.5% for SUVmax, 63.6% for SUVmean) than ^18^F-FDG (81.8% for SUVmax, 81.8% for SUVmean) in differentiating between breast cancer and benign breast lesions [43]. Thus, it remains difficult to clearly distinguish inflammatory pseudotumors from malignant lesions. Angiogenesis is an early pathological change in chronic inflammation, which may be the reason for the observed low specificity [8, 44], and neovascularization may vary depending on different stages of inflammation. Although we demonstrated an excellent specificity for tumor diagnosis in our meta-analysis, we cannot rule out the possibility of a lower specificity when using RGD PET/CT, especially in the early pathological stage of chronic inflammation.

In our meta-analysis, SUVmax from RGD PET/CT before CCRT may be able to predict short-term outcomes of treatment. In a study by Xiaohui Luan et al. addressing the ability of ^18^F-alfatide PET/CT to identify responders, the AUC of T/N in the lung (AUC=0.944) at baseline was higher than that of SUVmax, SUVpeak, T/N in the blood, and T/N in the muscle (AUC=0.815, 0.864, 0.889, and 0.901, respectively). In another study, SUVmax (AUC=0.737) before treatment and SUVmax (AUC=0.846), T/N (AUC=0.785) during treatment were also able to predict short-term outcomes, and SUVmax during treatment had superior predictive value compared to the volumetric parameters (AUC=0.786) of MRI. As a vital process in the growth and progression of tumors, reversal of angiogenesis may occur earlier than tumor cell death; however, FDG PET cannot detect it earlier. These findings may explain why RGDPET/CT can predict tumor treatment response [28, 45, 46], though neither Nadia Withofs et al. nor Xiaohui Luan et al. were able to identify responders using ^18^F-FDG PET/CT [27, 35, 46].

In mouse experiments, tumors with medium and high uptake of ^99^m-Tc-3P-RGD2 SPECT/CT before treatment responded well, with a greater degree of tumor response compared to tumors with low uptake levels [47]. In a pilot study with only four patients treated with bevacizumab-containing drugs (one patient with disease progression, one with a partial clinical response, and two with complete response), the patients exhibited different degrees of SUVmean decreases using ^18^F-FPPRGD2 PET/CT (1.6%, 7.9%, 25.2%, and 25.0%, respectively) [48]. In a study by Andrei Iagaru, patients with a 59.8% decrease in ^18^F-FPPRGD2 uptake had no recurrent GBM, whereas patients with a 4.8% decrease had recurrent disease [19]. RGD PET/CT may have potential for early prediction of the response to antiangiogenesis therapy, though these preliminary findings should be confirmed in larger studies.

There are limitations in this meta-analysis. First, expression of *α*v*β*3 varies in different types of tumors, which may affect the capability of RGD PET diagnostic and prediction. Second, the receptor binding affinity and receptor-binding kinetics of different radiotracers vary, which may also affect results. These limitations are all due to the limited number of studies. Therefore, more research with large sample sizes is urgently needed.

## 5. Conclusion

The interesting but preliminary data of this meta-analysis demonstrate that RGD PET/CT may be an ideal diagnostic method for detecting underlying malignancies in patients suspected of having tumors and may be able to predict short-term treatment outcomes. It is necessary to conduct large-scale clinical trials for RGD PET/CT to further study its diagnostic ability and predictive value for short-term outcomes.

## Figures and Tables

**Table 1:** Main characteristics of five studies for diagnosis of tumor included in this meta-analysis.

**Number**	**Study**	**Country**	**Year**	**Numbers of patients ** **(lesions)**	**Neoplasm**	**Sex ** **(M/F)**	**Mean age**	**Imaging**	**Study design**	**PET technique**	**Confirmation**
1	Andrei Iagaru	America	2014	8 (30)	Assessable breast cancer lesions	0/8	54.3±8.8	^18^F-FPPRGD2 PET/CT	Prospective	329.3MBq 60min	Histopathology

2	Song Gao	China	2015	26	Assessable lung cancer lesions	15/11	61.62±7.98	^18^F-alfatide RGD PET/CT	Prospective	213.34±29.8MBq 60min	Histopathology
3				16 (152)	Assessable lymph nodes	–	–				

4	Fei Kang	China	2015	34	Identify NSCLC from lung tuberculosis	19/15	42.4±15.6	^68^Ga-Alfatide II RGD PET/CT	Prospective	1.85 MBq/kg 60min	Histopathology
5				17	Assessable lymph nodes	–	–				

6	Kun Zheng	China	2015	91	Suspected lung lesions	48/43	56.5 ± 14.9	^68^Ga-NOTA-PRGD2 PET/CT	Prospective	111MBq 30min	Histopathology and follow-up
7				159	Assessable lymph nodes	–	–				

8	Yue Zhou	China	2017	13(196)	Assessable lymph nodes	6/7	57±12	^18^F-alfatide RGD PET/CT	Prospective	212.15±30.8MBq 60min	Histopathology

9	Jiang Wu	China	2018	44(53)	Assessable breast cancer lesions	0/44	50.73±8.01	^18^F-Alfatide II RGD PET/CT	Prospective	306 ± 80MBq 60min	Histopathology

M/F: the ratio of male to female.

**Table 2:** Results of RGD PET/CT for four parameters in the diagnosis of suspected carcinoma.

**Study**	**Year**	**Imaging**	**SUVmax**				**SUVmean**				**T/N**				**Visual**			
			**TP** **(Sen)**	**FP**	**FN**	**TN** **(Spe)**	**TP** **(Sen)**	**FP**	**FN**	**TN** **(Spe)**	**TP** **(Sen)**	**FP**	**FN**	**TN** **(Spe)**	**TP** **(Sen)**	**FP**	**FN**	**TN** **(Spe)**
Andrei Iagaru	2014	^18^F-FPPRGD2 PET/CT	22 (95.7%)	0	1	7 (100%)												

Song Gao_1_	2015	^18^F-alfatide RGD PET/CT	17 (100%)	5	0	4 (44.44%)												

Song Gao_2_	2015	^18^F-alfatide RGD PET/CT					13 (92.86%)	6	1	132 (95.65%)								

Fei Kang_1_	2015	^68^Ga-Alfatide II RGD PET/CT	16 (76.9%)	1	5	12 (90.48%)	18 (84.62%)	3	3	10 (76.19%)					18 (85.71%)	2	3	11 (84.62%)

Fei Kang_2_	2015	^68^Ga-Alfatide II RGD PET/CT	6 (75%)	0	2	9 (100%)												

Kun Zheng_1_	2015	^68^Ga-NOTA-PRGD2 PET/CT	55 (80.9%)	4	13	19 (82.6%)	57 (83.8%)	2	11	21 (91.3%)								

Kun Zheng_2_	2015	^68^Ga-NOTA-PRGD2 PET/CT	27 (77.14%)	3	8	121 (97.58%)												

Yue Zhou	2017	^18^F-alfatide RGD PET/CT	18 (90%)	7	2	169 (96%)	17 (85%)	14	3	162 (92.1%)	17 (85%)	7	3	169 (96%)	20 (100%)	9	0	167 (94.9%)

Jiang Wu	2018	^18^F-alfatide II RGD PET/CT	37 (88.1%)	5	5	6 (54.5%)	37 (88.1%)	5	5	6 (54.5%)					39 (92.9%)	4	3	7 (63.6%)

Subscript 1: the set of data for the diagnosis of carcinoma in situ. Subscript 2: the set of data for the diagnosis of metastasis. Sen: sensitivity; Spe: specificity. TP: true-positive, FP: false-positive, FN: false-negative, and TN: true-negative.

**Table 3:** Main characteristics of three studies for prediction of short-term outcome included in this meta-analysis.

**Study**	**Country**	**Year**	**Number of Patients**	**Neoplasm**	**Sex** **(M/F)**	**Mean Age**	**Imaging at Baseline**	**Study Design**	**Treatment**	**Response Criteria**
Nadia Withofs [26]	Belgium	2015	32	Locally advanced rectal cancer	23/9	63 ± 8	^18^F-FPRGD2 PET/CT	Prospective	CCRT	TRG (0 VS. 1-3)

Hui Zhang [27]	China	2015	25	GBM after surgical resection	15/10	49.5 ± 19.5	^18^F-alfatide RGD PET/CT	Prospective	CCRT	△VOLT_1-3_ (cutoff=58%)

Xiaohui Luan [28]	China	2016	18	Advanced NSCLC	14/4	62 ± 12.04	^18^F-alfatide RGD PET/CT	Prospective	CCRT	RECIST (CR, PR VS. SD, PD)

△VOLT_1-3_: the change of volume on MRI from baseline (T1) to the eleventh week (T3) after the start of CCRT. M/F: the ratio of male to female.

**Table 4:** Result of RGD PET/CT for SUVmax in the prediction of short-term outcomes.

**Study**	**Year**	**Imaging at Baseline**	**TP (Sen)**	**FP**	**FN**	**TN (Spe)**
Nadia Withofs	2015	^18^F-FPRGD2 PET/CT	5 (100%)	9	0	18 (66.7%)

Hui Zhang	2015	^18^F-alfatide RGD PET/CT	8 (72.7%)	3	3	11 (78.57%)

Xiaohui Luan	2016	^18^F-alfatide RGD PET/CT	7 (77.8%)	1	2	8 (88.9%)

Sen: sensitivity; Spe: specificity. TP: true-positive, FP: false-positive, FN: false-negative, and TN: true-negative.

**Table 5:** The Newcastle-Ottawa Scale was used to assess the quality of the studies for prediction.

	Selection				Comparability	Outcome			Quality score
Study	Representativeness of the exposed cohort	Selection of the nonexposed cohort	Ascertainment of exposure	Demonstration that outcome of interest was not present at start of study	Comparability of cohorts on the basis of the design or analysis	Assessment of outcome	Was follow-up long enough for outcomes to occur	Adequacy of follow-up of cohorts	
Nadia Withofs et al	*✓*	*✓*	*✓*	*✓*	*✓*	*✓*	*✓*	*✓*	8

Hui Zhang et al	*✓*	*✓*	*✓*	*✓*	*✓*		*✓*	*✓*	7

Xiaohui Luan et al	*✓*	*✓*	*✓*	*✓*	*✓*		*✓*	*✓*	7

*✓* means the score in the term.

**Table 6:** Chi-square test was used to assess heterogeneity among the included studies and Spearman correlation was used to assess the threshold effect among the studies.

**Study**	**Parameter**	**Sensitivity**				**Specificity**				**Spearman correlation**	
		**χ** ^2^	**p**	**I** ^**2**^	**Heterogeneity**	**χ** ^2^	**p**	**I** ^**2**^	**Heterogeneity**	**Coefficient**	**P**
Diagnostic value	SUVmax	13.00	0.0722	46.1%	Moderate	40.34	0.0000	82.6%	High	0.405	0.320
	SUVmean	1.08	0.8970	0.00%	Low	16.81	0.0021	76.2%	High	-0.100	0.873

Predictive value	SUVmax	2.59	0.2733	22.90%	Low	2.11	0.3488	5.10%	Low	0.500	0.667

**χ** ^2^: Chi-square, I^2^: inconsistency (I-sequence); p: p value.

**Table 7:** Subgroup analysis for SUVmax and SUVmean for the diagnostic value of RGD PET/CT.

	SUVmax			SUVmean	
Factors	Primary or metastatic lesions	RGD radioligands	Tumor types	RGD radioligands	Primary or metastatic lesions

P-value	0.1612	0.1214	0.8209	0.2018	0.1882
